# Supplementary material for: Spectral phase control of interfering chirped pulses for high-energy narrowband terahertz generation
Source: Nat Commun. 2019 Jun 13;10:2591. doi: 10.1038/s41467-019-10657-4 (PMC6565633; doi:10.1038/s41467-019-10657-4)
Supplement: Supplementary file 1 — Supplementary Information [file 41467_2019_10657_MOESM1_ESM.pdf]

Spectral Phase Control of Interfering Chirped Pulses for High-Energy Narrowband Terahertz Generation

Supplementary Information

Spencer W. Jolly[1, 2, †, *], Nicholas H. Matlis[3, †], Frederike Ahr[3], Vincent Leroux[1, 2], Timo Eichner[1], Anne-Laure Calendron[3, 4], Hideki Ishizuki[5, 6], Takunori Taira[5, 6], Franz X. Kärtner[3, 4], and Andreas R. Maier[1]

[1] Center for Free-Electron Laser Science and Department of Physics Universität Hamburg, Luruper Chaussee 149, 22761 Hamburg, Germany

[2] Institute of Physics of the ASCR, ELI-Beamlines project, Na Slovance 2, 18221 Prague, Czech Republic

[3] Center for Free-Electron Laser Science and Deutsches Elektronen Synchrotron (DESY), Notkestraße 85, 22607 Hamburg, Germany

[4] Department of Physics and The Hamburg Centre for Ultrafast Imaging, Universität Hamburg, Luruper Chaussee 149, 22761 Hamburg, Germany

[5] Division of Research Innovation and Collaboration, Institute for Molecular Science, 38 Nishigonaka, Myodaiji, Okazaki, Aichi 444-8585, Japan

[6] Innovative Light Sources Division, RIKEN SPring-8 Center, 1-1-1 Kouto, Sayo-cha, Sayo-gun, Hyogo 679-5148 Japan

† authors contributed equally to this work

* corresponding author: spencer.jolly@cea.fr

Supplementary Note 1: Spectral Phase Optimisation at 558 GHz

To verify our concept of maximising THz output by spectral phase manipulation, we repeat the measurements shown for 361 GHz in the main text with a large-aperture, magnesium-doped PPLN (LA-PPMgLN) crystal of 558 GHz output.

Supplementary Figure 1a shows the THz signal as a function of delay between the driver pulses, using a crystal with 212 µm poling period, which corresponds to a THz frequency of 558 GHz. With no GDD or TOD compensation applied (blue triangles), THz is generated with low signal levels over a broad range of delays. Each delay corresponds to matching the instantaneous frequency difference, $\Delta\omega$, to the LA-PPMgLN frequency Ω_{THz} at a temporally and spectrally distinct part of the pulse. Since the difference frequency required to drive THz at 558 GHz is larger, compared to the 361 GHz crystal, THz generation occurs at later delays, consistent with our predicted Δt (see Methods). Gradually adding GDD to one of the two pulses enhances the THz signal and narrows the range of THz-generating delays. The experimentally determined optimum of ΔGDD = 12,940 fs^2 or 69 mm SF11, is in very good agreement with our calculation (see Methods) predicting ΔGDD = 14,170 fs^2. At the optimum ΔGDD the THz signal is increased by a factor of 6.5.

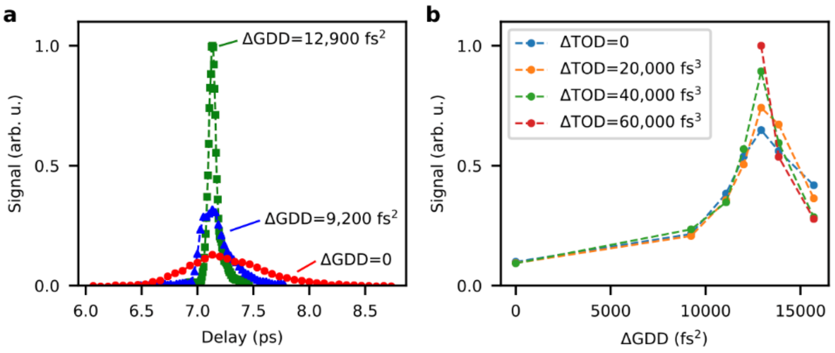

Supplementary Figure 1 | Spectral Phase Manipulation for a 558 GHz LA-PPMgLN: Pulse delay scan matching Δω to the THz frequency of the LA-PPMgLN crystal (blue: uncompensated driver pulses). Adding ΔGDD, the THz signal increases and the range of THz-generating delays narrows. (b) Adding ΔTOD at the optimum ΔGDD, the signal increases by 55%, yielding a total increase in THz output by a factor of 10.

Incrementally adding third order phase, Supplementary Figure 1b, the THz signal is further increased by 55%. The experimentally determined optimum of ΔTOD = 60,000 fs^3 (three DCMs) is close to the calculated optimum of ΔTOD = 83,519 fs^3. We attribute this difference to contributions to the presence of uncompensated fourth and higher order phases in the driver pulse.

Supplementary Figure 2 shows the THz output at various delays, both positive and negative, with no added ΔGDD (red), and with ΔGDD = 9,200 fs^2 or 49 mm SF11 (green). These results show the increase in peak THz output and narrowing of the delay behaviour already seen. In contrast, we show a decrease in peak THz output and widening of the delay behaviour at negative delays. These results agree with results shown at 361 GHz in Figure 4b of the main text and confirm the conceptual picture of the compensation of the chirp curvature. The results also agree with calculations shown as solid lines.

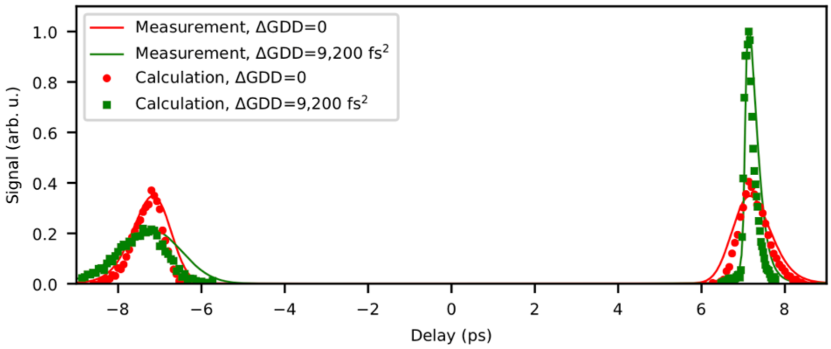

Supplementary Figure 2 | Delay Scan Behaviour for 558 GHz LA-PPMgLN: The delay scan behaviour at both positive and negative delays confirms the concept detailed in the main text. At the positive delay the added ΔGDD improves the peak output and narrows the range of delays which generate THz, as already shown. At negative delays the added ΔGDD has the opposite effect, decreasing the peak signal and increasing the range of delays that generate THz.

Using the 558 GHz crystal with optimised spectral phase (ΔGDD = 12,900 fs^2, ΔTOD = 60,000 fs^3) we vary the input fluence to determine the conversion efficiency of the driver laser into THz, shown in Supplementary Figure 3. We obtain an average THz energy of 128 μJ, pumping with a fluence of 186 mJ/cm^2. The lower peak *extracted* conversion efficiency of 0.07% is attributed to a combination of factors. The first is a narrower phase-matched THz bandwidth (due to the larger number of poling cycles, i.e., 170 cycles for the 212 μm period vs. 109 cycles for the 330 μm period) which increases the sensitivity of the process to TOD and higher order phase that are responsible for variations in $\Delta\omega$. The reduction in efficiency (0.64×) due to this effect is to first order inversely proportional to the number of periods. The second factor is the quadratic dependence on THz frequency inherent to the DFG process which yields a factor of 2.4×. The third factor is due to the increase in absorption coefficient with THz frequency in lithium niobate [1, 2] which affects the efficiency quadratically and accounts for a factor of ~0.3×. The net result is a reduction of the *extracted efficiency* of 0.46×, i.e. 0.07% for 558 GHz compared to 0.15% for 361 GHz, which is very close to the

measured ratio of efficiencies of the two crystals. Please compare the main manuscript for our definition of "extracted" and "intrinsic" efficiency.

Because 558 GHz is close to an absorption peak in air, primarily due to the relative humidity [3], the setup was purged with dry nitrogen to remove the effect of absorption.

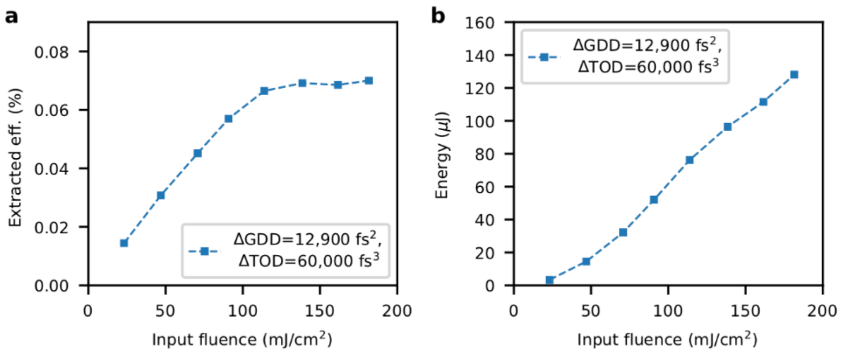

Supplementary Figure 3 | Conversion Efficiency: (a) Extracted efficiency for increasing driver fluence, measured with a 212 µm poling period (558 GHz) LA-PPMgLN crystal and optimised spectral phase (ΔGDD = 12,900 fs^2, ΔTOD = 60,000 fs^3). The conversion efficiency saturates around 0.07%. (b) We obtain an average pulse energy of 128 µJ pumping with 186 mJ/cm^2.

Supplementary Note 2: Frequency Measurement

Supplementary Figure 4 shows the characterisation of the THz frequency, generated by the 330 μm and 212 μm poling period LA-PPMgLN crystals. The measurements were performed with an interferometric correlation using metallic mirrors and a silicon beam splitter. The THz central frequency of 361 GHz (330 μm) and 558 GHz (212 μm) is in very good agreement with theory, predicting 370 GHz and 574 GHz, respectively.

Corresponding to the comment in the caption of Figure 5, the measurements (blue dots) in Supplementary Figure 4 are averages of ten measurements, and the sine fits shown emphasise the high precision of the frequency measurement. Since the sampling, especially in the 361 GHz case, is a large fraction of the central frequency the fluctuations can visually seem to have a larger effect than in reality. Finally, a closer look of the calculated spectrum shown in Figure 5d of the main text is presented in Supplementary Figure 4, showing less than 3.5 GHz FWHM bandwidth, or less than 1% of the central frequency.

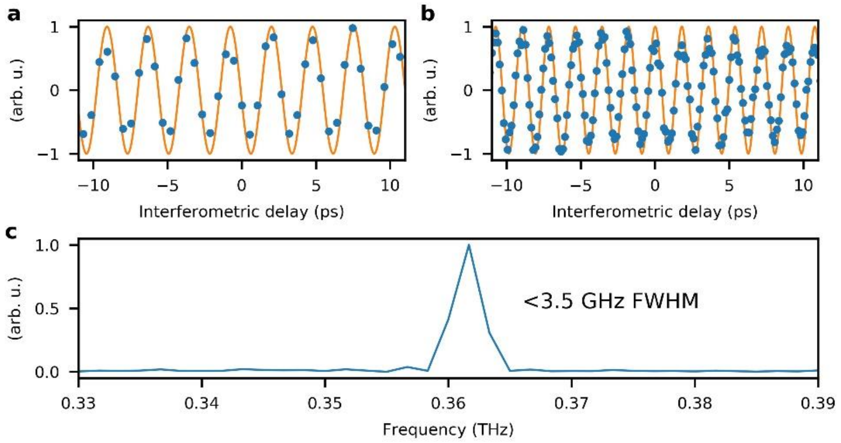

Supplementary Figure 4 | Frequency Characterisation: Interferometric correlation of the THz signal for the 330 μm poling period PPLN crystal (a), and 212 μm poling period PPLN crystal (b), yielding THz central frequencies of 361 GHz and 558 GHz in very good agreement with theoretical predictions of 370 GHz and 574 GHz, respectively. Sine fits are shown, emphasising the very good agreement. Panel (c) is a closer look at the retrieved frequency of the full trace for the 330 μm shown in Fig. 5d of the main text.

Supplementary Note 3: Internal Conversion Efficiency at 361 GHz

Supplementary Figure 5 shows the internal conversion efficiency of the optimally GDD and TOD corrected driver pulse using the 330 µm (361 GHz) crystal. It corresponds to the extracted conversion efficiency shown in Fig. 5a of the main text.

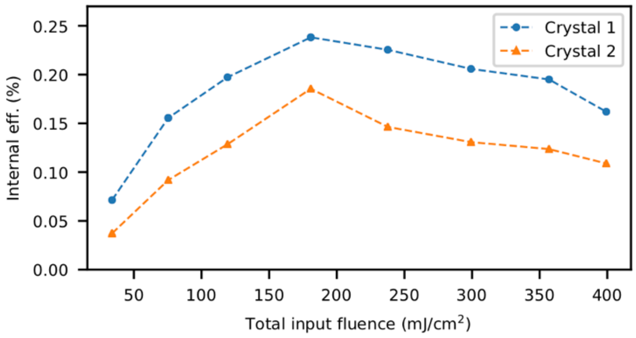

Supplementary Figure 5 | Internal Conversion Efficiency: Conversion efficiency of the optimally GDD and TOD corrected driver pulse shown in Fig. 5a of the main text using the definition of an internal conversion efficiency.

Supplementary Note 4: Regularisation of the pulse train in intensity

As is referenced in the main text, the effect of the TOD on chirped-and-delayed pulse copies is equivalently an effect on the bandwidth of the frequency content and an effect on the regularity of the produced pulse train in intensity. For the application of THz generation, the effect on the inherent difference-frequency content is more instructive as shown in Fig. 4a of the main text, but for other applications the effect specifically on the pulse train may be more relevant. Supplementary Figure 6 shows an example of this effect on the pulse train, calculated for a 120 GHz difference frequency (Δt = 1.55 ps), and having a larger TOD by a factor of 4 than that in the main results. The larger TOD and lower matched frequency are chosen to more easily visualise the effect. It is clear that with the compensation of the TOD the pulse train has a more regular spacing, which corresponds also to a narrower difference-frequency content.

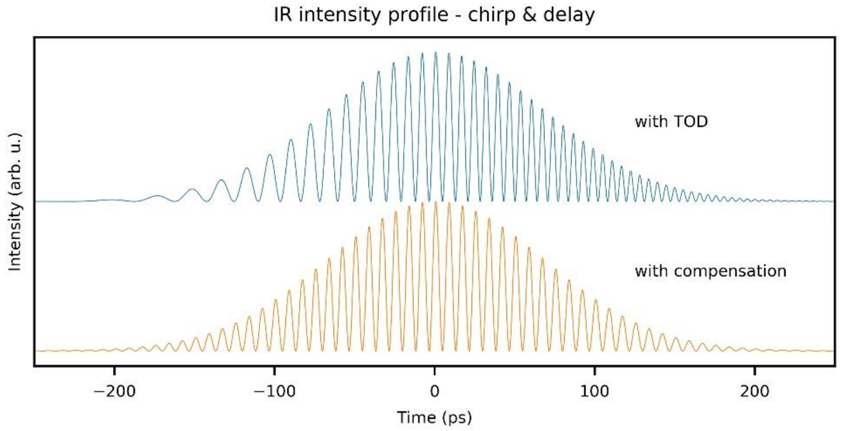

Supplementary Figure 6 | Pulse Train: The effect of TOD and compensation of TOD on the pulse train in the intensity of the overlapped chirped pulses. The pulse train with TOD on the driving pulses has a varying spacing (top, blue line), and the pulse train with compensation has a regularised spacing (bottom, orange line). In this figure the overlapped pulses are matched for 120 GHz difference frequency, with a TOD 4x larger than in the main text in order to more clearly show the effect.

Supplementary Note 5: Variation of the pulse train structure

Fig. 4a of the main text shows, that as the chirp compensation is implemented, the difference frequency content is contained more within the narrow bandwidth of the quasi-phase matching process in the long PPLN crystals. Equivalently, this can be viewed as the pulse train periodicity becoming more regular, as depicted in Supplementary Figure 6.

Supplemental Figure 7 illustrates this principle using experimental data shown in Fig. 3a of the main text. As we add ΔGDD and ΔTOD the pulse train regularizes. Increasingly, a larger fraction of the pulse train matches the narrow bandwidth of the crystal and engages in THz generation.

In general, the spacing of pulses in the pulse train is proportional to the inverse of the difference frequency. With careful tuning and the addition of ΔTOD the pulse train structure could be tailored to a desired application.

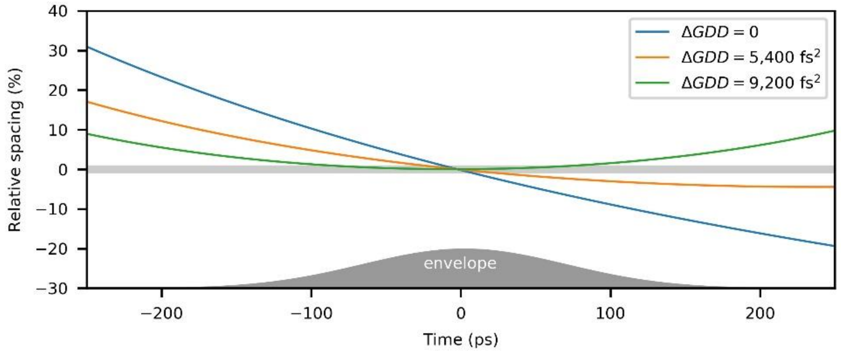

Supplementary Figure 7 | Pulse Train Variation: The periodicity (spacing) of the pulse train in the IR beam intensity, shown relative to the central spacing of 2.78 ps (for 361 GHz), for different levels of chirp compensation. The amounts of ΔGDD shown correspond to those used in the terahertz generation experiment, Fig 3a., with the 1% bandwidth shown in light grey.

Supplementary References

[1] Unferdorben, M., Szaller, Z., Hajdara, I., Hebling, J. & Pálfalvi, L. Measurement of refractive index and absorption coefficient of congruent and stoichiometric lithium niobate in the terahertz range. *Journal of Infrared, Millimeter, and Terahertz Waves* **36,** 1203-1209 (2015).

[2] Wu, X., Zhou, C., Huang, W. R., Ahr, F., & Kärtner, F. X. Temperature dependent refractive index and absorption coefficient of congruent lithium niobate crystals in the terahertz range. *Opt. Express* **23,** 29729-29737 (2015).

[3] Slocum, D. M., Slingerland, E. J., Giles, R. H., & Goyette, T. M. Atmospheric absorption of terahertz radiation and water vapor continuum effects. *Journal of Quantitative Spectroscopy and Radiative Transfer* **127,** 49-63 (2013).
